# Metastatic dedifferentiated liposarcoma of the mediastinum with osteosarcomatous differentiation: A case report

**DOI:** 10.1097/MD.0000000000040748

**Published:** 2024-12-06

**Authors:** Cong Hu, Shuxiong Nong, Aihua Liu, Weiling Huang, Yin Qi, Ying Wang, Yingfang Ye, Yuancheng Jiang, Yixin Chen, Qi Zhang, Meng Wu

**Affiliations:** a Department of Ultrasound, Zhongnan Hospital of Wuhan University, Wuhan, Hubei, China; b Department of Cardiology, Baise People’s Hospital. Affiliated Southwest Hospital of Youjiang Medical University for Nationalities, Baise, Guangxi, China; c Department of Ultrasound, The Sixth Hospital of Wuhan, Affiliated Hospital of Jianghan University, Wuhan, Hubei, China; d Teaching Office, Zhongnan Hospital of Wuhan University, Wuhan, Hubei, China; e Department of Ultrasound, Hanchuan People’s Hospital of Hubei Province, Xiaogan, Hubei, China; f Department of Pediatrics, Xianning Central Hospital, The First Affiliated Hospital of Hubei University of Science and Technology, Xianning, Hubei, China.

**Keywords:** dedifferentiated, liposarcoma, mediastinum, osteosarcomatous differentiation

## Abstract

**Rationale:**

Liposarcoma is a malignant tumor of adipocytic differentiation that rarely arises within the mediastinum. Most of the existing data available comes from scattered case reports and a few small series.

**Patient concerns:**

A 51-year-old man was admitted with cough and sputum accompanied by fever. X-ray and CT showed a bulky anterior mediastinal mass that was initially misdiagnosed as a teratoma. PET/CT demonstrated a lesion on that location showing a small area of moderately intense uptake. After surgical excision, histopathological examination via hematoxylin and eosin first revealed the diagnosis of malignant undifferentiated tumors. The results of immunohistochemical evaluations were as follows: P63 (scattered +), VIMENTIN (giant cell +), CD68 (KPI; giant cell +), SMA (−), and Ki-67 (Li: 50%). Molecular pathology: MDM2 gene status (+) with amplification.

**Diagnoses:**

The final histopathological diagnosis was dedifferentiated liposarcoma with osteosarcomatous differentiation.

**Interventions:**

The patient underwent left mediastinal tumor resection, left upper lobe wedge resection and postoperative chemotherapy.

**Outcomes:**

Mediastinal recurrences and chest wall metastases occurred quickly before the second round of chemotherapy 2 months later. Four months after surgery, the patient died.

**Lessons:**

This paper presents a case of dedifferentiated liposarcoma with osteosarcomatous differentiation in a rare location: the mediastinum. Correct diagnosis is of importance for appropriate choice of therapy. Clinicians should be aware of the presence of a dedifferentiated liposarcoma within a mass on the mediastinum and enhancing treatment and management strategies for affected patients.

## 1. Introduction

Liposarcoma (LPS) is a malignant tumor composed of adipogenic cells with varying degrees of differentiation and heterogeneity, accounting for approximately 20% of soft tissue sarcomas.^[1]^ LPS can be divided according to intermediate local invasive and malignant biological behaviors. The former refers to atypical lipomatous tumor/highly differentiated liposarcoma (ALT/WDLPS), while the latter includes dedifferentiated liposarcoma (DDLPS), myxoid liposarcoma, pleomorphic liposarcoma, and nonspecific liposarcoma. DDLPS is a rare subtype of LPS. In this paper, we present a rare case of DDLPS with osteosarcomatous differentiation of the mediastinum in a male.

Dedifferentiated liposarcoma (DDL) with osteosarcoma-like differentiation is a rare and aggressive cancer that signifies a transformation of a liposarcoma into a tumor with additional osteosarcoma-like traits. A key genomic feature of this tumor is the amplification of the MDM2 gene, which is a crucial aid in its diagnosis.^[2–4]^ The standard treatment approach typically involves surgical resection, complemented by chemotherapy and radiation therapy as necessary. The significance of such cases is highlighted by their rarity and the potential for these tumors to manifest in various anatomical locations, even sometimes resembling kidney tumors. For instance, a 60-year-old patient admitted for a presumed left kidney tumor was found to have a tumor in an unexpected location during surgery.^[3]^ The tumor was successfully removed using laparoscopic techniques, and the amplification of the MDM2 gene was confirmed. Remarkably, the patient remained free of recurrence a year and a half postsurgery, even though the surgery did not involve a margin of healthy tissue.^[3]^ Precise immunohistochemical and molecular studies are vital for impacting the efficacy and prognosis of further treatments. Moreover, the prognosis for DDL with osteosarcoma-like differentiation is often grimmer than other liposarcoma subtypes, due to the tumor’s aggressive nature and metastatic potential, which compound the challenges in effectively managing the disease.

Reports of these cases underscore the diagnostic and therapeutic challenges, emphasizing the importance of accurate histopathological assessment, immunohistochemistry, and molecular diagnostics, as well as the need for vigilant postoperative care.^[3]^ For managing such complex cases, it is essential that patients are treated at specialized sarcoma centers with expertise in handling these intricate scenarios. In summary, cases of dedifferentiated liposarcoma with osteosarcoma-like differentiation hold significant value for medical research, diagnostic challenges, treatment strategies, and prognostic evaluations. By sharing the case report, medical professionals can gain a deeper understanding of the complexities of this rare disease, thereby enhancing treatment and management strategies for affected patients.

## 2. Case description

**Figure 1.**
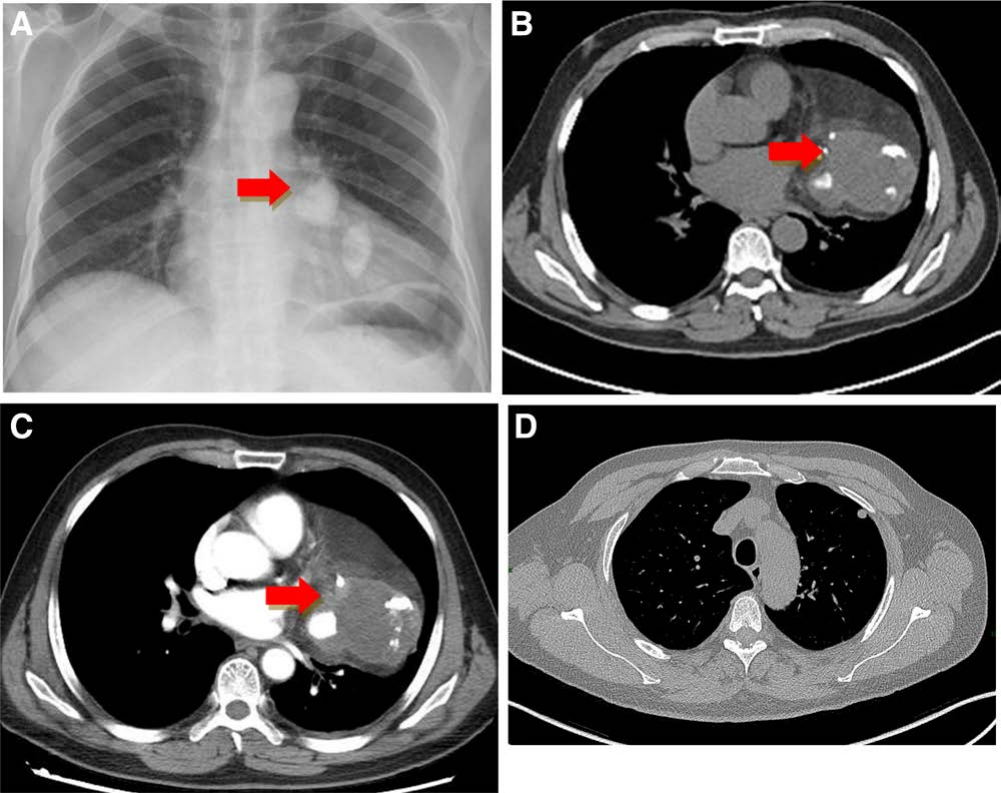
Chest X ray and CT. (A) Chest radiograph showing a high-density shadow. Regular (B) and contrast-enhanced (C) views of a chest CT scan demonstrating a heterogeneous mass (13.9 cm × 8.4 cm × 8.1 cm) in the anterior mediastinum, containing soft tissue (asterisks), fat, peripheral calcification, and low-attenuation areas of probable necrosis. A capsule is present; it is incomplete and insufficient to contain the tumor. (D) A solid nodule of the left upper lobe. CT = computed tomography.

**Figure 2.**
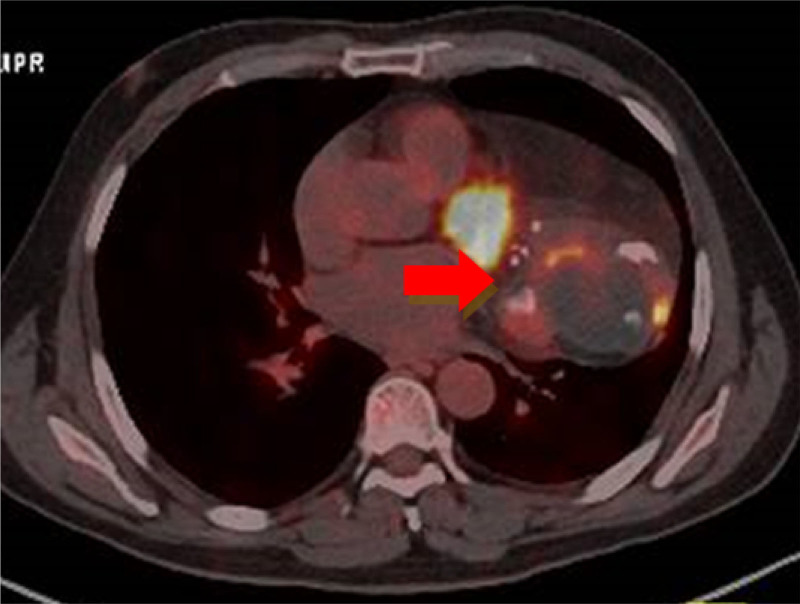
PET. PET revealed FDG accumulation in the tumor. FDG = Fluorodeoxyglucose, PET = positron emission tomography.

A 51-year-old man presented with complaints of cough, dyspnea, and fever for 7 days. He was previously treated as a case of pituitary neuroendocrine tumors presumptively, and surgery for the removal of a pituitary tumor was performed through craniotomy 5 years ago. The patient (BMI: 26.8) was a current smoker (smoking about 5 cigarettes a day) and moderate drinker. Apart from a history of pituitary neuroendocrine tumors, the patient declared having no medical comorbidities and no family medical history. Chest X-ray showed 2 circular high-density shadows, and there was a soft tissue shadow in the left lower lung overlapping the left heart (Fig. 1A). Computed tomography (CT) showed a large heterogeneous mediastinal mass involving the middle mediastinum with well-defined lateral margins within the left lung and indistinct medial margins within the mediastinum. The mass showed coarse, chunky calcification, large fatty and soft tissue components, enhanced septa within (Fig. 1B, C) and a solid nodule in the upper lobe of the left lung (Fig. 1D). Additional imaging studies, including bone scan and cranial and abdominal CT, showed no secondary lesions elsewhere. A positron emission tomography/computed tomography (PET/CT) scan showed most of the mass to be non-FDG-avid, with a small area of moderately intense uptake (SUVmax = 18.8) in the lateral portion (Fig. 2). The solid nodule in the upper lobe of the left lung showed a slightly intense uptake (SUVmax = 5.2). Tracer distribution in the hilar and mediastinal nodal stations, liver, adrenal glands, bone marrow and other organs was within normal limits and thus not suggestive of any alternative primary site.

**Figure 3.**
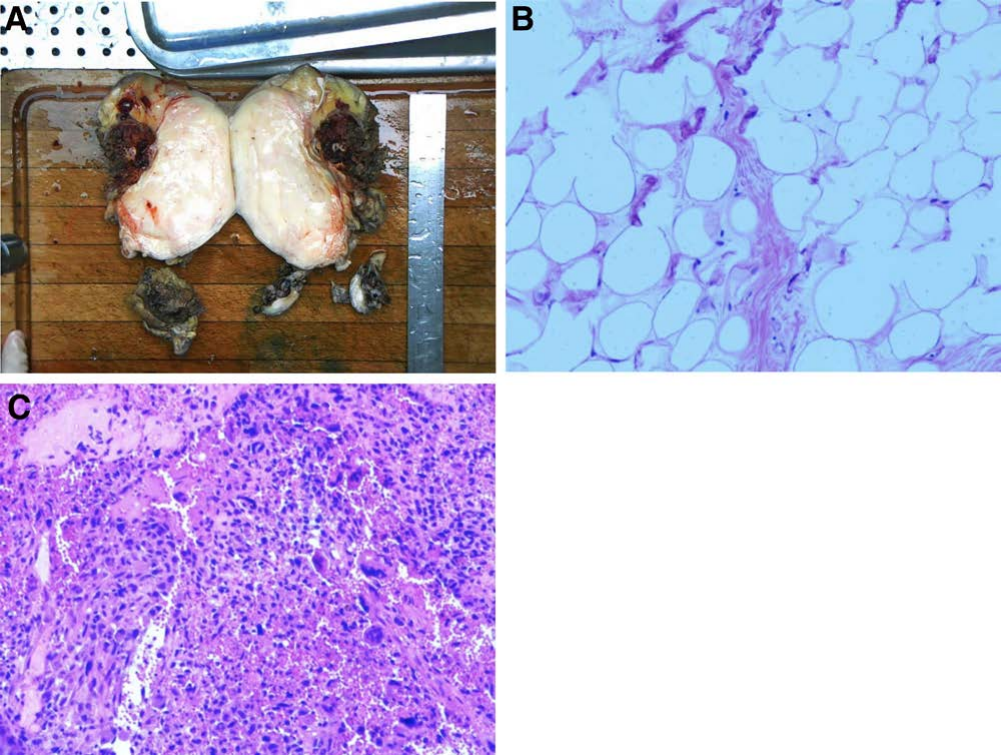
Histological findings. (A) a tumor with a diameter of approximately 1 cm could be reached (left thoracic tumor). (B) The differentiation components are mainly short fusiform cells under the microscope, and nuclear division is mostly seen, containing many large nuclear giant cells. (C) The nodules in the upper lobe of the left lung were metastatic tumor nodules, which look the same as thoracic tumors under the microscope. (HE) × 100.

**Figure 4.**
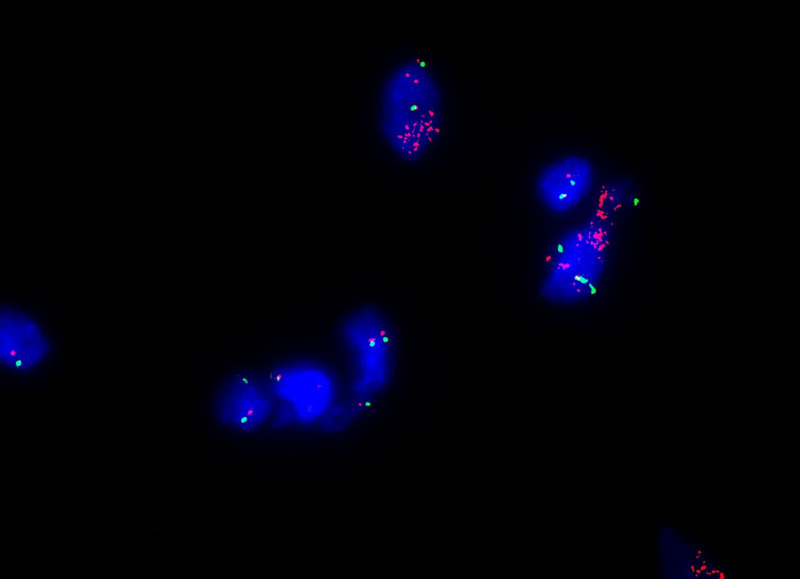
The diagnosis of DDLPS was confirmed by fluorescence in situ hybridization (FISH), showing MDM2 amplification. DDLPS = dedifferentiated liposarcoma, FISH = fluorescence in situ hybridization.

The patient underwent left mediastinal tumor resection and left upper lobe wedge resection. During the intraoperative exploration of the upper lobe of the left lung, a tumor with a diameter of approximately 1 cm could be reached under the pleura in front of the left lung, and a 13 × 10 × 10 cm mass could be seen at the left border of the heart. The capsule was still intact, and the basement was closely adhered to the left upper pulmonary vein, pulmonary trunk and pericardium. An incision revealed that the tumor contained brown bean curd-like residue, fat, and dental contents (Fig. 3A). Postoperative pathology showed the following. Soft tissue tumor (left thoracic tumor); spindle cell components showed hemorrhage, a large number of multicellular giant cells, tissue cells, and small vascular hyperplasia. In addition, some well-differentiated adipose tissue and deformed necrotic osteoid tissue were observed. Tumor tissues could be seen in the tissues (nodules in the upper lobe of the left lung; Fig. 3B). Pathological examination in our hospital showed a (mediastinal) giant cell tumor of soft tissue with ossification and lung metastasis, mediastinal atypical fat tumor/highly differentiated liposarcoma dedifferentiated, with the components of dedifferentiation being giant cell tumor of soft tissue with lung metastasis (Fig. 3C). Immunohistochemistry showed the following: P63 (scattered +), VIMENTIN (giant cell +), CD68 (KPI; giant cell +), SMA (−), and Ki-67 (Li: 50%). Further assessment at the Fudan University Shanghai Cancer Center demonstrated differentiated liposarcoma (on the left side of the chest tumor), with the following composition: rich in osteoclastic-like giant broken bone malignant tumors, giant cell tumors in most of the regions, local tumor tissue formation, hints of the giant cell type of osteosarcoma, and tumor metastasis to regional lymph nodes. The molecular pathology was as follows: MDM2 gene status (+), with amplification (Fig. 4). The nodules (pulmonary nodules) were metastatic tumor nodules with the same microscopic appearance as thoracic tumors. The mediastinal mass and the left upper lobe lesion pathologically were same, primary site: mediastinum. The patient underwent left mediastinal tumor resection and left upper lobe wedge resection and postoperative chemotherapy: intensified doxorubicin (75 mg/m^2^; 25 mg/m^2^ per day, days 1–3) plus ifosfamide (10 g/m^2^ over 4 days with mesna and pegfilgrastim). However, mediastinal recurrences and chest wall metastases occurred quickly before the second round of chemotherapy 2 months later. Four months after surgery, the patient died.

## 3. Discussion

DDLPS accounts for approximately 18% of LPS, which was proposed by Evans in 1979. Most DDLPSs are primary tumors (80% ~ 90%), and secondary dedifferentiation can occur in 10% of cases, most of which are related to multiple recurrences of WDLPS.^[5,6]^ DDLPS mostly occurs in middle-aged and elderly patients, with no significant sex difference. The onset sites are diverse, most commonly the retroperitoneum, and can also occur in the mediastinum, thorax, abdomen, pelvic cavity, trunk, limbs, groin, and skin.^[7,8]^ The most common lesion in this group was retroperitoneum. Clinical symptoms show a bump that increases gradually and is painless; more often, incidental discovery occurs or a bump in the viscera brings about corresponding symptoms. The clinical diagnosis is difficult due to delayed symptoms and a lack of specificity.

Our case of dedifferentiated liposarcoma with osteosarcomatous dedifferentiation in the mediastinum represents an extremely rare entity with. The pathological diagnosis of DDLPS requires the presence of 2 components in the same tumor, namely, WDLPS and cellular-rich non-liposarcoma components. MFH is the most common of the dedifferentiated components, but other sarcomas can also be seen, especially in the dedifferentiated area, such as some rare histological images resembling cancer, melanoma, meningioma, lymphoma, or angiosarcoma. In this case, there are 2 components and heterogeneous components of well-differentiated WDLPS and dedifferentiated non-fatty polymorphic undifferentiated sarcoma. Immunohistochemical markers were used to determine the diagnosis of mediastinal dedifferentiated liposarcoma with heterogeneous giant cell osteosarcoma differentiation. This study, as a case report, has its inherent limitations. Case reports are typically based on a single or a small number of cases, which limits the generalizability and external validity of the findings. Due to the small sample size, they may not represent a broader patient population, leading to potential biases. Case reports often lack a control group, making it difficult to exclude the confounding effects of other non-study factors.

Treatment of dedifferentiated liposarcoma with osteosarcomatous differentiation usually involves a multidisciplinary approach. Improving the prognosis of this patient requires comprehensive consideration of multiple factors and treatment methods. In our patient, surgical resection is the main method for treating liposarcoma. Adjuvant chemotherapy was recommended after surgery to reduce the risk of recurrence and improve outcomes. Outpatient follow-up with regular, appropriate diagnostics is critical. With a deeper understanding of the biological characteristics and molecular changes of liposarcoma, future treatment methods may focus more on developing personalized treatment plans, including targeted drugs and immunotherapy. For example, Alisterib, as a targeted drug, is currently being studied for its therapeutic efficacy in patients with advanced or metastatic sarcoma.

## Author contributions

**Conceptualization:** Cong Hu, Aihua Liu, Ying Wang, Yuancheng Jiang, Yixin Chen, Meng Wu.

**Data curation:** Cong Hu, Shuxiong Nong, Aihua Liu, Yuancheng Jiang, Meng Wu.

**Formal analysis:** Aihua Liu, Ying Wang, Yingfang Ye, Meng Wu.

**Investigation:** Yin Qi, Yixin Chen.

**Methodology:** Cong Hu, Shuxiong Nong, Qi Zhang.

**Resources:** Qi Zhang, Meng Wu.

**Software:** Shuxiong Nong, Yin Qi, Qi Zhang, Meng Wu, Cong Hu.

**Supervision:** Meng Wu.

**Writing – original draft:** Meng Wu, Cong Hu.

**Writing – review & editing:** Shuxiong Nong, Weiling Huang, Meng Wu, Cong Hu.
